# Proteomics analysis of the matrisome from MC38 experimental mouse liver metastases

**DOI:** 10.1152/ajpgi.00014.2019

**Published:** 2019-09-23

**Authors:** Arseniy E. Yuzhalin, Su Yin Lim, Alex N. Gordon-Weeks, Roman Fischer, Benedikt M. Kessler, Dihua Yu, Ruth J. Muschel

**Affiliations:** ^1^Cancer Research United Kingdom/Medical Research Council Oxford Institute for Radiation Oncology, Department of Oncology, University of Oxford, Oxford, United Kingdom; ^2^Research Institute for Complex Issues of Cardiovascular Diseases, Kemerovo, Russia; ^3^Department of Biomedical Sciences, Faculty of Medicine and Health Sciences, Macquarie University, Sydney, Australia; ^4^Nuffield Department of Surgical Sciences, University of Oxford, John Radcliffe Hospital, Oxford, United Kingdom; ^5^Target Discovery Institute, Nuffield Department of Medicine, University of Oxford, Oxford, United Kingdom; ^6^Department of Molecular and Cellular Oncology, The University of Texas MD Anderson Cancer Center, Houston, Texas

**Keywords:** annexin A1, colorectal cancer, extracellular matrix, liver metastasis, matrisome, S100-A11

## Abstract

Dissemination of primary tumors to distant anatomical sites has a substantial negative impact on patient prognosis. The liver is a common site for metastases from colorectal cancer, and patients with hepatic metastases have generally much shorter survival, raising a need to develop and implement novel strategies for targeting metastatic disease. The extracellular matrix (ECM) is a meshwork of highly crosslinked, insoluble high-molecular-mass proteins maintaining tissue integrity and establishing cell–cell interactions. Emerging evidence identifies the importance of the ECM in cancer cell migration, invasion, intravasation, and metastasis. Here, we isolated the ECM from MC38 mouse liver metastases using our optimized method of mild detergent solubilization followed by biochemical enrichment. The matrices were subjected to label-free quantitative mass spectrometry analysis, revealing proteins highly abundant in the metastatic matrisome. The resulting list of proteins upregulated in the ECM significantly predicted survival in patients with colorectal cancer but not other cancers with strong involvement of the ECM component. One of the proteins upregulated in liver metastatic ECM, annexin A1, was not previously studied in the context of cancer-associated matrisome. Here, we show that annexin A1 was markedly upregulated in colon cancer cell lines compared with cancer cells of other origin and also over-represented in human primary colorectal lesions, as well as hepatic metastases, compared with their adjacent healthy tissue counterparts. In conclusion, our study provides a comprehensive ECM characterization of MC38 experimental liver metastases and proposes annexin A1 as a putative target for this disease.

**NEW & NOTEWORTHY** Here, the authors provide an extensive proteomics characterization of murine colorectal cancer liver metastasis matrisome (the ensemble of all extracellular matrix molecules). The findings presented in this study may enable identification of therapeutic targets or biomarkers of hepatic metastases.

## Graphical abstract

**Figure:**
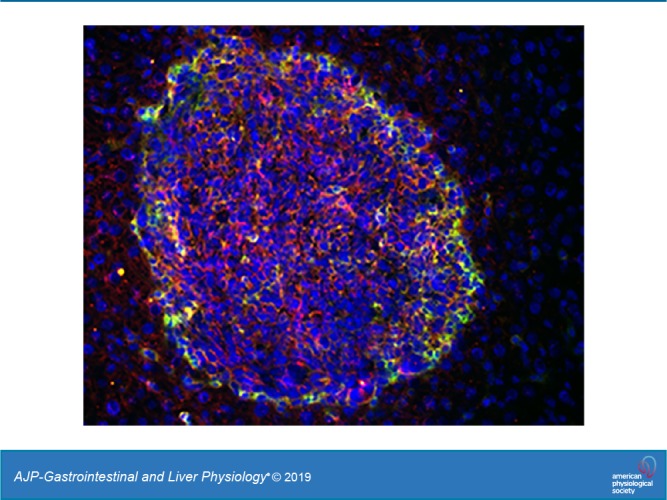

## INTRODUCTION

The liver is a frequent site for metastases from colorectal cancer, and despite that survival from this disease has substantially improved in the last several decades, the management of hepatic metastases is still difficult (27, 33). The main available curative option for patients with hepatic metastases is surgery; nonetheless, subjects undergoing surgical intervention can expect a 5-yr overall survival of ~40–50%, with up to 10% alive after 10 years (20, 30, 40). As such, more basic research is needed to broaden our knowledge about the biological basis of liver metastasis and advance current treatment modalities for this disease.

The extracellular matrix (ECM), composing the bulk of tumor stroma, has a leading role in progression of many cancers, including colorectal adenocarcinoma (6, 60a), and has been suggested to facilitate metastasis formation (60). Alterations in the matrisome (i.e., the ensemble of ECM proteins) enable tumor cells to invade surrounding tissues and intravasate into capillaries through multiple mechanisms, including promotion of cell proliferation (28), invasion (25), and triggering of the epithelial-to-mesenchymal transition (47). Despite significant progress to unravel the significance of the ECM remodeling in tumor biology (29), its impact on the liver metastatic milieu remains undefined.

Annexin A1 is a phospholipid-binding, Ca^2+^-dependent protein regulating cell behavior by inhibiting autophagy (63), enhancing inflammation and apoptosis (14, 63), suppressing proliferation (43), etc. In the ECM, conflicting reports implicate annexin A1 in both improving fibroblast synthetic activity (26) and exerting antifibrotic effects (38), potentially suggestive of a context-dependent function for this molecule. Even though annexin A1 was found to be relevant for development of several cancer types (4, 19, 52), no coherent paradigm for its role currently exists in the literature, and it is unknown whether this protein is a driver or passenger in the process of tumor progression.

Here, we used a decellularization approach, described by our group previously (59), followed by high-throughput proteomics to characterize comprehensively the ECM derived from experimental murine colon cancer hepatic metastases. We identified a list of 13 significantly upregulated matrisome proteins that collectively predicted poor prognosis in patients with colorectal adenocarcinoma but not other cancers characterized by a substantial ECM contribution (i.e., notable for its extensive stromal component). With the use of this unbiased method, we further examined annexin A1 as a putative therapeutic target for colorectal cancer by comprehensively characterizing its expression levels, tissue localization, cellular source in the tumor microenvironment (TME), and potential role in the ECM milieu.

## METHODS

### 

#### Ethics.

Human colorectal cancer liver metastasis tissues and surrounding, uninvolved hepatic tissues were obtained from the Oxford Radcliffe Biobank. Approval from the local ethical committee was granted following the full institutional review (Ethics Number 09/H0606/5). This study complied with the Declaration of Helsinki. Informed consent was obtained from all patients.

#### Animal studies.

Animal studies were conducted in accordance with the United Kingdom Animal (Scientific Procedures) Act 1986, as well as guidelines for animal welfare (55). Animal studies were performed within the limits of the project license issued by the United Kingdom Home Office (PPL 30/2841 and 30/3413). Female, aged severe combined immunodeficiency mice were purchased from ENVIGO (Bicester, UK); female C57BL/6 mice were bought from Charles River Laboratories (Kent, UK). To generate murine liver metastases, mice were anesthetized using vaporized isoflurane, and the upper lateral abdominal wall was incised with the following injection of 1 × 10^6^ HT-29 or 5 × 10^5^ MC38 cells, prepared in 100 µL PBS, into the splenic parenchyma. The spleen was excised ~1 min postinjection to prevent growth of splenic tumors. The wound was then closed using nonsoluble sutures and autoclips. Control mice have been given a mock surgery to account for surgical stress. Severe combined immunodeficiency mice injected with human HT-29 cells were humanely culled ~35 days after surgery, and C57BL/6 mice injected with MC38 cells were euthanized ~13 days after surgery. For subcutaneous injection, 1 × 10^6^ Lewis lung carcinoma (LLC) cells were injected into the flank of C57BL/6 mice. Subcutaneous tumor volume was assessed by caliper measurements of tumor height, length, and width. Multiplication of values obtained for three dimensions was used to calculate tumor volume.

#### Cell lines.

Mouse colon cancer cell line MC38, mouse LLC, and human colon cancer cell line HT-29 were used in the study. MC38 cells were purchased from Kerafast; HT-29 and LLC cells were bought from American Type Culture Collection. Early passage cancer cells were cultured at 37°C and 5% CO_2_ in either Roswell Park Memorial Institute-1640 media (R8758; Sigma) or DMEM (D2429; Sigma), supplemented with 10% FBS (16000-044; Thermo Fisher Scientific), and 100 IU/mL penicillin-streptomycin (15140-122; Thermo Fisher Scientific).

#### Liver decellularization.

Excised mouse livers (intact or metastasis bearing) were washed with PBS once and placed in a dish containing 1% SDS and 0.01% ammonium hydroxide (or NH_4_OH) in double-distilled (dd)H_2_O. The dishes were placed on a shaker for 72 h. Decellularization buffer was changed every 6 h during the day, and dishes were left at −4°C overnight.

#### ECM enrichment and mass spectrometry.

Decellularized livers were cut into small 100-mg pieces, placed into 200 µL ice-cold buffer C of the CNMCs Compartmental Protein Extraction Kit (K3013010; BioChain Institute), and homogenized using the blade homogenizer. After brief sonication, 3,000–4,000 units of peptide *N*-glycosidase F (or PNGase F; P0704; New England BioLabs) and 1 μL Benzonase (E1014; Sigma) were added into samples following the incubation for 1 h at 4°C. Samples were spun down at 18,000 *g* for 20 min. The supernatant was removed, and the pellet was washed in 400 μL ice-cold buffer W of the CNMCs Compartmental Protein Extraction Kit at 4°C for 5 min. The protein extract was then spun at 18,000 *g* for 20 min. The supernatant was discarded, and the pellet was resuspended in 150 μL ice-cold buffer N and incubated at 4°C for 20 min to solubilize nuclear proteins. Protein extract was spun at 18,000 *g* for 20 min. The supernatant was discarded, and the pellet was resuspended in 150 μL ice-cold buffer M for solubilization of membrane-bound proteins. The extracts were then spun down at 18,000 *g* for 20 min, and the supernatant was discarded. The remaining pellet was resuspended in 150 μL prewarmed buffer and incubated at room temperature for 20 min to solubilize cytoskeletal proteins. Protein extract was spun at 18,000 *g* for 20 min. The supernatant was discarded, and the pellet was resuspended in 150 μL buffer C, incubated at 4°C for 5 min, and then spun again for 20 min at 15,000 *g* at 4°C. The resultant insoluble pellet, consisting of ECM proteins, was snap frozen and stored at −20°C until use.

Samples were then solubilized in a mixture of 8M urea (9U5378; Sigma), 100 mM ammonium bicarbonate (09830; Sigma), and 10 mM DTT (43817; Sigma), pH 7.8, and incubated at 37°C for 30 min. Iodoacetamide (I1149; Sigma) was added to a final concentration of 25 mM, and the samples were further incubated for 30 min at room temperature in the dark. Protein concentration was then measured by the detergent-compatible protein assay (5000111; Bio-Rad). Protein was then precipitated via the methanol-chloroform technique with the following resuspension in 50 μL 8M urea and vortexing. Eventually, the urea concentration was reduced to a final concentration of <1M by dilution of the mixture with ddH_2_O. ECM-enriched pellets were digested overnight using trypsin (V5111; Promega) at a ratio of 1:50 enzyme/substrate. Samples were constantly vortexed on a shaker at 37°C. Another portion of trypsin was added at a ratio of 1:100 enzyme/substrate, and samples were again incubated for 4–6 h at 37°C while being vortexed.

Protein samples were then analyzed with nanoliquid chromatography tandem mass spectrometry (nano-LC-MS/MS), using the Acquity LC instrument (C18 column with a 75-µm × 250-mm, 1.7-µm particle size; Nanoacquity Waters), coupled to a Thermo LTQ Orbitrap Elite mass spectrometer (resolution of 120,000 at 400 mass-to-charge ratio, Top 20, collision-induced dissociation), using a gradient of 1–35% acetonitrile for 60 min at a flow rate of 250 nL/min. Peak lists of MS/MS spectra were generated using MSConvert (Proteome Wizard) and further searched using Mascot version 2.3 (http://www.matrixscience.com) against the Swiss-Prot protein database containing mouse (16,642 entries as of September 2012) or human (20,306 entries as of June 2014) sequences with tryptic restriction and mass deviations of 10 parts per million/0.5 Da in the respective MS modes. Oxidation of methionine, deamidation of asparagine and glutamine, and other known collagen and proteoglycan modifications were used as variable modifications. Peptide false discovery rate was adjusted to 1%. For label-free quantification of differentially expressed proteins, normalized abundance of each protein was determined by the measurement of the peak area intensity using Progenesis QI software (Nonlinear Dynamics). Briefly, protein abundance was calculated from the sum of all unique peptide ion abundances for a specific protein on each run. Normalization of abundance was performed to allow comparisons across different sample runs by the software. Proteins identified by more than one peptide were retained. The normalized peptide intensities for each sample were used to calculate fold-change ratios for proteins between sample groups.

The MS proteomics data have been deposited to the ProteomeXchange consortium via the Proteomics Identifications (or PRIDE) partner repository with the data set identifiers PXD013350 and 10.6019/PXD013350.

#### Silver staining.

Equal amounts of protein lysates were mixed with NuPAGE LDS Sample Buffer (NP0008; Thermo Fisher Scientific). Proteins were separated by use of SDS-PAGE at 100 V for 2 h. Silver staining was performed using the SilverQuest staining kit (LC6070; Life Technologies), according to the manufacturer’s instructions.

#### Tissue staining.

Excised or decellularized tissues were embedded in the optimal cutting temperature compound (Tissue-Tek; VWR), snap frozen, and stored at −80°C until use. Tissue sections were cut using the OTF 5000 cryostat (Bright Instruments). For immunostaining, the sections were briefly fixed in acetone, washed with PBS, blocked with 20% goat/donkey serum, and incubated overnight with the following primary antibodies: annexin A1 (AF3770; R&D Systems), S100-A11 (10237-1-AP; Proteintech), collagen IV (ab6586; Abcam), collagen V (ab7046; Abcam), fibronectin (ab2413; Abcam), laminin (ab30320; Abcam), CD11b (ab62817; Abcam), Ly6G (551459; BD Biosciences), and CD3 (ab33429; Abcam). The next day, sections were briefly rinsed in PBS with the following incubation with secondary antibodies for 1–1.5 h at room temperature. The slides were washed again and mounted using the ProLong Diamond Antifade Mountant with 4′,6-diamidino-2-phenylindole (DAPI; P36962; Thermo Fisher Scientific) before being imaged using an epifluorescence (DM IRB/E; Leica) or confocal (LSM880; Zeiss) microscope. Hematoxylin and eosin staining was performed using a standard protocol.

#### Immunoblotting.

Equal amounts of protein lysates were mixed with NuPAGE LDS Sample Buffer. Proteins were separated using the SDS-PAGE method at 150 V for 1 h and transferred at 30 V for 1 h at 4°C on the polyvinylidene fluoride membrane (IPVH00010; Millipore) with the following block in 5% skim milk, diluted in Tris-buffered saline with 0.05% Tween-20 (TBST; P7949; Sigma). Blots were incubated for 18 h at 4°C with one of the following primary antibodies: annexin A1 (AF3770; R&D Systems), actin (sc-47778; Santa Cruz Biotechnology), or GAPDH (D16H11; Cell Signaling Technology). Membranes were washed thrice in TBST, followed by the addition of horseradish peroxidase-conjugated secondary antibodies (all Amersham), and incubation for 1 h at room temperature. Membranes were washed thrice in TBST, and protein bands were visualized with enhanced chemiluminescence using an X-ray detector.

#### ELISA.

Annexin A1 concentration in serum was determined by the MyBioSource (MBS166640) ELISA kit in accordance with the manufacturer’s instructions.

#### Cell transfection.

Confluent LLC cells (70–80%) were washed with PBS once and transfected with lentivirus particles harboring shRNA targeting the *Anxa1* gene (TRCN0000109728; Sigma) or vector scramble control diluted in Opti-MEM reduced serum medium (31985062; Thermo Fisher Scientific). Opti-MEM was replaced with complete media, 24 h posttransfection, and cells were further cultured with addition of 2 μg/mL puromycin to eliminate untransfected cells.

#### RNA extraction and PCR.

Total RNA was isolated from cultured cells using TRIzol reagent (15596026; Thermo Fisher Scientific) in accordance with the manufacturer’s instructions. The quality and quantity of RNA were determined using a spectrophotometer NanoDrop 3300 (Thermo Fisher Scientific). Contaminating DNA was removed using the TURBO DNA-free Kit (AM1907; Thermo Fisher Scientific). RNA was converted to complementary DNA using the High-Capacity RNA-to-cDNA Kit (4387406; Thermo Fisher Scientific). Resulting cDNA was mixed with pre-designed forward and reverse KiCq Start primers (Sigma-Aldrich) in the presence of Power SYBR Green PCR Master Mix (4367659; Thermo Fisher Scientific). PCR was performed using a Stratagene MX3005p PCR machine.

#### Proliferation assay.

Cells (1 × 10^3^) were seeded in five, 96-well plates. Six technical replicates were used per condition. At consecutive time points, separated 24 h apart, complete media were replaced with fresh serum-free media containing 10% WST-1 viability and proliferation reagent (ab155902; Abcam), and cells were incubated for an additional 45–60 min. Absorbance was read at 450 nm using the plate reader.

#### Migration assay.

For migration assay, 24 mm Corning Transwell polycarbonate membrane cell culture inserts were used (CLS3428-24EA; Sigma). Cells (1 × 10^5^) in 200 µL serum-free media were seeded into Transwell inserts with 8.0 µm pores. Six technical replicates were used per condition. Cells were allowed 15–20 min to settle, and then 300 µL complete media was added into lower chambers to stimulate cell migration through pores. After 48 h culture, Transwell inserts were discarded, and migrated cells in lower chambers were formalin fixed with the following staining with 0.05% crystal violet diluted in 10% formalin. Crystal violet was washed twice with ddH_2_O, and cells were then visualized using a bright-field microscope with a digital camera (Nikon). ImageJ software was used to quantify density of migrated cells in lower chambers.

#### Bioinformatics.

Categorization of matrisome proteins was performed in accordance with the study by Naba et al. (35). Proteins were divided into the following groups: proteoglycans, glycoproteins, collagens, ECM regulators, ECM-affiliated proteins, and secreted factors. The PANTHER bioinformatics resource (34) (http://pantherdb.org/) was used to perform a Gene Ontology term-enrichment analysis. To investigate the role of protein alteration on cancer survival and prognosis, as well as coexpression of annexin A1 and S100-A11, the cBioPortal bioinformatics resource was used (7). For analysis of annexin A1 expression in colorectal carcinoma and normal colon tissues, Oncomine bioinformatics software (45) was used. The Broad Institute’s Cancer Cell Line Encyclopedia (https://portals.broadinstitute.org/ccle) was used to analyze coexpression of annexin A1 and S100-A11 in 1,072 cancer cell lines.

#### Statistical analysis.

For analysis of two groups with unpaired samples, Mann-Whitney *U* test was used. For analysis of two groups with paired samples, Wilcoxon signed-rank test was used. For analysis of more than three groups, Kruskal-Wallis test with Dunn’s multiple comparison posttest was used. Spearman rank correlation was used to determine the coexpression between annexin A1 and S100-A11. To identify differences between groups in the quantitative proteomics analysis, tumor growth curves, or cell line growth, two-way ANOVA was used. For comparison of survival curves, the log rank test was used. The false discovery rate was used to validate peptide and protein hits obtained during the quantitative LC-MS/MS analysis. Values with *P* < 0.05 were considered statistically significant.

## RESULTS

### 

#### Generation of mouse MC38 liver metastasis followed by isolation and enrichment of its ECM.

**Fig. 1.**
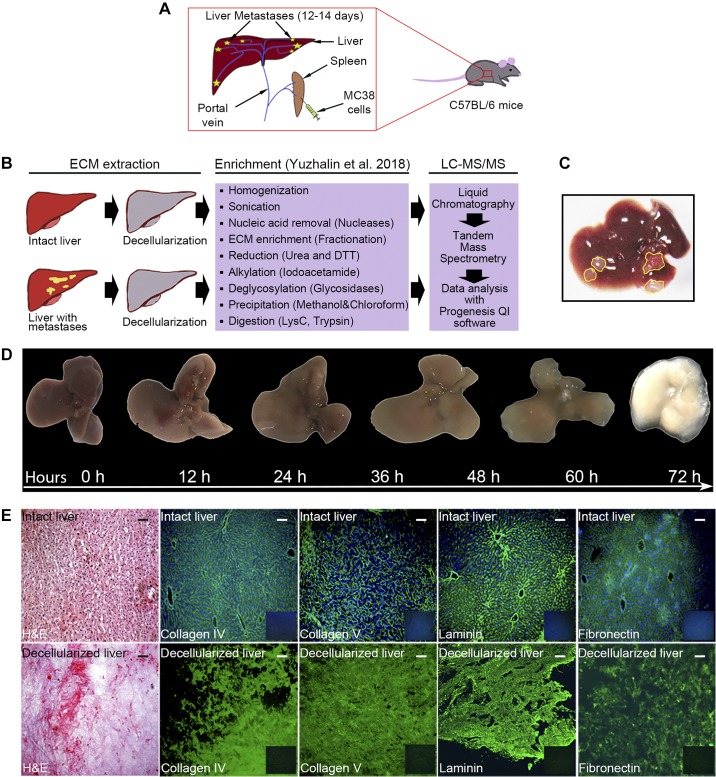
Study design and preparation of decellularized matrices for proteomics analysis. *A*: a cartoon illustrating the model for experimental mouse liver metastases. Briefly, MC38 mouse colon cancer cells were injected into the spleen parenchyma, and tumor cells traveled to the liver via the portal vein. Splenectomy was performed to exclude cancer cell formation in the splenic bed. Mice were humanely culled, and hepatic metastases were excised 12–14 days postoperation. *B*: study workflow describing extracellular matrix (ECM) isolation and enrichment protocol with the following label-free proteomics analysis [Yuzhalin et al. (59)]. *C*: representative image of a mouse liver bearing MC38 metastasis. Metastatic lesions are outlined in yellow. *D*: representative images of mouse livers during decellularization over 72 h. *E*: decellularized or intact mouse livers were cryosectioned and stained for the indicated ECM proteins (green) or with hematoxylin and eosin (H&E). All sections were counterstained with 4′,6-diamidino-2-phenylindole (DAPI; blue). Original scale bars, 100 μm. Isotype control staining is provided in bottom-right corners. LC-MS/MS, liquid chromatography tandem mass spectrometry.

With the aim of the investigation of the proteome of murine liver metastasis ECM, we first generated experimental hepatic metastases using a well-characterized MC38 colorectal cancer cell line injected intrasplenically into B16/C57 mice (Fig. 1*A*). Isolation and clean-up of the ECM is challenging because its hydrophobicity and extensive crosslinking. We used a matrix decellularization and enrichment protocol developed by others (35) and modified by our group (59) (Fig. 1*B*).

Approximately 13 days postintrasplenic injection of MC38 cells, multiple (two to eight) metastatic foci developed in the livers of animals (Fig. 1*C*). As a control, we analyzed tumor-free livers from mice subjected to mock surgery to account for the potential effect of surgical stress on the ECM. We did not analyze uninvolved tissues adjacent to the metastasis site, because such specimens cannot be considered as adequate controls due to recognized reprogramming of these tissues by host cells, including ECM alterations caused by cancer-associated fibroblasts and other stromal cells affected by cancer cells (2, 13).

Livers were extracted from euthanized mice, and metastases were immediately excised using a scalpel. Tissues (metastatic lesions and unaffected parenchyma from mock-operated mice) were decellularized in a cocktail of detergents and further biochemically enriched by removal of contaminating cellular fractions of proteins, nucleic acids, and oligosaccharides. An example of mouse liver decellularization is presented in Fig. 1*D*. Decellularized tissue scaffolds retained the ECM architecture and morphology, as demonstrated by staining for characteristic matrix proteins collagens IV and V, laminin, and fibronectin (Fig. 1*E*). The decellularized tissues were positively stained for eosin (preferentially stains protein), whereas hematoxylin, which preferentially stains nucleic acids, was greatly diminished, indicating that nuclei and cytoplasmic RNAs had mainly been removed (Fig. 1*E*).

**Fig. 2.**
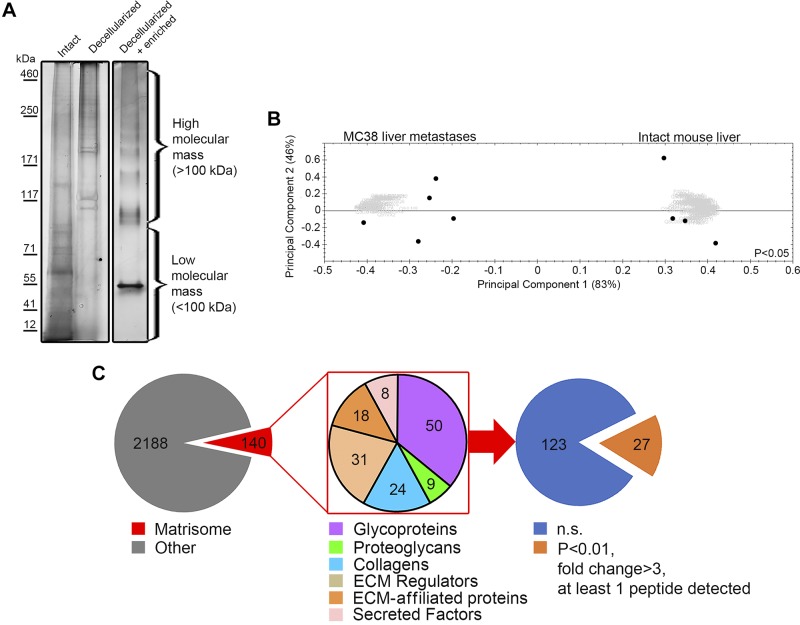
Label-free quantitative proteomics of intact and MC38 metastasis-bearing mouse livers. *A*: total protein was isolated from intact (*left*), decellularized (middle), and decellularized plus biochemically enriched (*right*) mouse livers and resolved by SDS-PAGE with the following silver staining. High and low molecular mass areas were considered <100 kDa and >100 kDa, respectively. *B*: intact livers or MC38 metastasis dissected from mouse livers were decellularized and extracellular matrix (ECM) enriched. After analysis by liquid chromatography tandem mass spectrometry with label-free quantitation (*n* = 3 biological replicates), the principal component analysis of relative protein abundances between metastasis and intact livers was computed (*P* < 0.05). Percentage of variance is displayed in parentheses. Gray “clouds” indicate individual proteins, whereas black circles represent replicates. *C*: proteomics analysis revealed 2,328 proteins in the ECM (both groups were considered). Of these, 140 proteins were classified as the matrisome in accordance with the categorization proposed by Naba et al. (35). Twenty-seven of 140 proteins were significantly different between groups (two-way ANOVA, *P* < 0 0.01) after restriction to a fold-change threshold of >3 and identification of at least 1 peptide. n.s., not significant.

After subcellular fractionation and enzymatic depletion of DNA, RNA, and sugars, the successful enrichment of samples for ECM proteins was confirmed by SDS-PAGE separation with the following silver staining of protein bands (Fig. 2*A*). The increased proportion of high molecular mass proteins in samples testified to the abundance of matrix proteins, because the ECM is predominantly composed of high molecular mass proteins (>100 kDa), many of which are formed in the extracellular compartment after generation of smaller protein chains within the cell. As a result, we generated high-purity, ECM-rich preparations from MC38 mouse liver metastasis.

#### Label-free MS analysis of resultant matrices.

Matrices generated using this method or similar protocols are generally suitable for high-throughput MS analysis (35, 37, 59). To gain understanding of the proteomic difference between murine metastatic and intact ECM, we performed a label-free MS analysis of normal and metastatic matrices (*n* = 3 biological replicates per group). The principal component analysis revealed a distinctive ECM composition of tumor lesions compared with the normal hepatic tissue (Fig. 2*B*). The proteomics identified 2,328 proteins in preparations, of which 140 were classified as matrisomal, in accordance with categorization established in a seminal study by Naba et al. (35) (Fig. 2*C*; Supplemental Information^1^; see https://doi.org/10.17504/protocols.io.w9cfh2w). The proportion of contaminating non-ECM proteins was similar to that of similar studies (35, 59) and can be explained by a substantial number of cellular proteins being bound to the ECM during decellularization, as well as some proteins not yet being identified as matrisomal.

**Fig. 3.**
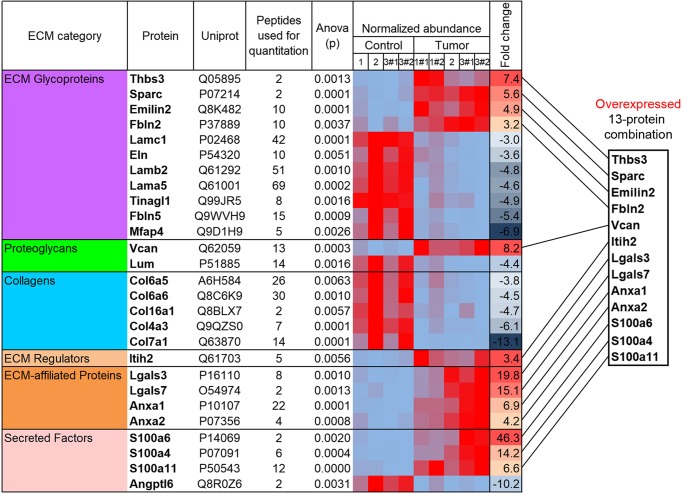
A heat map displaying 27 extracellular matrix (ECM) proteins with significantly different abundance between intact and MC38 metastasis-bearing mouse livers (two-way ANOVA, *P* < 0.01). These proteins were selected based on identification of at least 1 peptide and a fold-change threshold of >3 and ranked in accordance with their corresponding ECM category and fold change. #Technical replicate.

With the application of a threshold fold change of greater than three, 27 differentially expressed proteins between intact and MC38-derived hepatic metastases tissues were identified (Fig. 2*C*) and plotted as a heat map (Fig. 3; *P* < 0.01). Of these, 14 proteins were designated as substantially downregulated (suggesting that they may be specific hepatic ECM proteins), whereas 13 hits were dramatically upregulated. Technical replicates displayed a high consistency in protein abundance indicative of a high-quality analysis (Fig. 3). Classification of these 27 proteins using the PANTHER software (34) revealed a strong over-representation of categories associated with the ECM, thereby providing an in silico confirmation of successful ECM enrichment of LC-MS/MS-analyzed samples (Table 1).

**Table 1.** Significantly different liver metastasis proteins (27 hits) were subjected to PANTHER over-representation test for molecular function and cellular component

Molecular Function	Background Frequency	Sample Frequency	Expected	Fold Enrichment	Over-represented (+) or Under-represented (−)?	*P* Value
*Molecular Function (Gene Ontology)*						
Extracellular matrix structural constituent conferring tensile strength	38	7	0.04	>100	+	1.06E-10
Extracellular matrix structural constituent	139	21	0.16	>100	+	8.14E-39
Structural molecule activity	726	22	0.82	26.98	+	1.56E-26
Integrin binding	122	6	0.14	43.79	+	1.40E-05
Cell adhesion molecule binding	229	6	0.26	23.33	+	5.24E-04
*Cellular Component (Gene Ontology)*						
Laminin-10 complex	3	3	0.00	>100	+	5.01E-05
Laminin complex	9	4	0.01	>100	+	2.00E-06
Basement membrane	105	10	0.13	75.72	+	1.54E-13
Collagen-containing extracellular matrix	352	26	0.44	58.73	+	2.01E-41
Extracellular matrix	473	27	0.59	45.38	+	5.74E-41
Extracellular region part	2,156	27	2.71	9.96	+	1.77E-23
Extracellular region	2,717	28	3.42	8.19	+	4.27E-23
Extracellular matrix component	48	11	0.06	>100	+	4.88E-19
Collagen type I trimer	2	2	0.00	>100	+	1.29E-02
Fibrillar collagen trimer	11	4	0.01	>100	+	3.81E-06
Banded collagen fibril	11	4	0.01	>100	+	3.81E-06
Supramolecular polymer	879	8	1.11	7.24	+	1.32E-02
Supramolecular complex	880	8	1.11	7.23	+	1.33E-02
Complex of collagen trimers	16	6	0.02	>100	+	2.31E-10
Collagen trimer	81	10	0.10	98.16	+	1.35E-14
Laminin-1 complex	3	2	0.00	>100	+	2.15E-02
Collagen type IV trimer	6	3	0.01	>100	+	2.10E-04
Network-forming collagen trimer	6	3	0.01	>100	+	2.10E-04
Collagen network	6	3	0.01	>100	+	2.10E-04
Basement membrane collagen trimer	6	3	0.01	>100	+	2.10E-04
Synaptic cleft	20	4	0.03	>100	+	2.94E-05
Neuromuscular junction	98	4	0.12	32.45	+	1.10E-02
Extracellular space	1,914	24	2.41	9.97	+	6.27E-19

Displayed only Bonferroni-corrected results (*P* < 0.05).

**Fig. 4.**
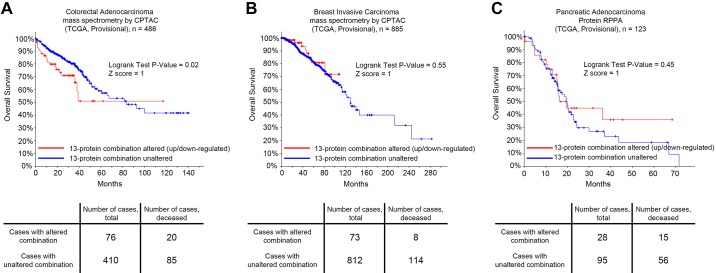
Overall survival of patients with colorectal adenocarcinoma (*A*), breast-invasive carcinoma (*B*), and pancreatic adenocarcinoma (*C*) who had an alteration in the 13-protein combination provided in Fig. 3 (alteration here means significant overexpression or underexpression). Log rank test. CPTAC, Clinical Proteomic Tumor Analysis Consortium; RPPA, reverse-phase protein array; TCGA, Tissue Cancer Genome Atlas.

Because proteins downregulated in metastasis ECM are likely to be liver-specific matrix molecules (therefore, probably unrelated to tumor development), we then specifically focused on the 13-protein combination overexpressed in murine liver metastasis ECM (Fig. 3). We then questioned if alterations in the expression of a resulting 13-protein combination can be clinically relevant in terms of cancer prognosis. To address this, we used the cBioPortal for Cancer Genomics software, enabling us to track survival of cancer patients in different modes of gene or protein expression. Strikingly, a combination of 13 proteins significantly predicted overall survival from colorectal adenocarcinoma based on assessment of 486 patients (Fig. 4*A*; *P* = 0.02). However, when we considered other malignancies with a recognized role of the ECM component, such as pancreatic adenocarcinoma (21, 53) or invasive breast cancer (31), no significant differences in survival rates were detected (Fig. 4, *B* and *C*). Hence, these findings suggest that the obtained 13-protein ECM combination is specifically relevant for progression of colorectal cancer.

#### Expression of annexin A1 in colorectal cancer liver metastases.

We then became particularly interested in annexin A1, an ECM-affiliated molecule that was consistently overexpressed in the murine metastatic tissue by almost sevenfold, which was represented by 22 individual peptides and displayed the highest significance value (*P* < 0.0001). This protein was previously linked to promoting 5-fluorouracil resistance (39), as well as inhibiting NF-κB (62) in colon cancer cells; however, its involvement in the ECM biology has not been described so far.

**Fig. 5.**
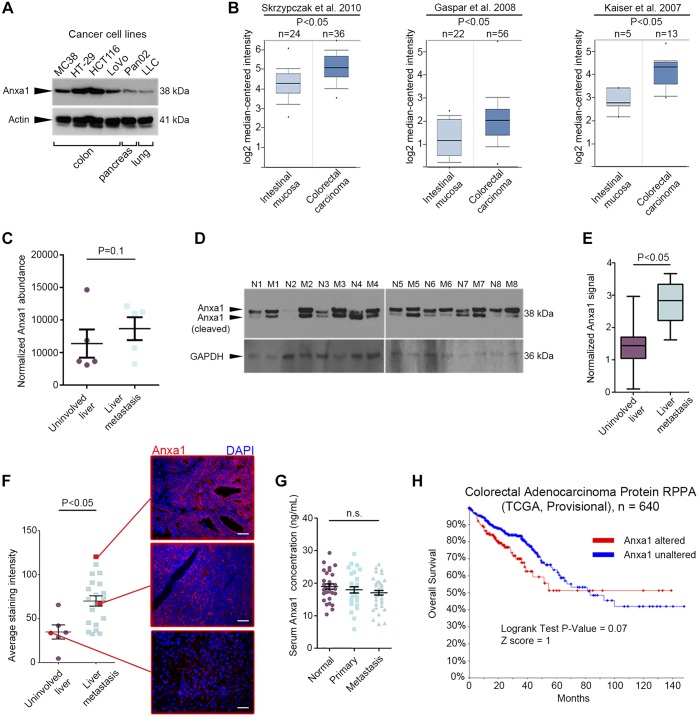
Annexin A1 is overexpressed in the extracellular matrix (ECM) from colorectal cancer liver metastases and might serve as a useful prognostication marker. *A*: annexin A1 expression in different cancer cell lines. *B*: Oncomine bioinformatics analysis of 3 different studies [see Skrzypczak et al. (47a) (*left*), Gaspar et al. (15a) (*middle*), and Kaiser et al. (23a) (*right*)] evaluating annexin A1 expression in patients with colorectal cancer. Mann-Whitney test. Small circles indicate range, error bars indicate median, whiskers indicate 95% confidence interval, box bounds indicate 25th and 75th quartiles. *C*: normalized annexin A1 abundance in the decellularized and enriched ECM fraction from colorectal cancer liver metastases or adjacent unaffected liver tissues (*n* = 5 per group). Circles and squares indicate biological replicates, error bars indicate mean, whiskers indicate SE. Extracted from Yuzhalin et al. (59). *D*: immunoblotting for annexin A1 in 8 resected colorectal cancer liver metastases (labeled as M) and adjacent unaffected liver tissue specimens (labeled as N). Whole tissue lysate was used for immunoblotting. GAPDH was used as a loading control. *E*: densitometry analysis of bands from the experiment in *D*. Wilcoxon signed-rank test. Error bars indicate median, whiskers indicate range, box bounds indicate 25th and 75th quartiles. *F*: average staining intensity (*left*) and representative microphotographs (*right*) of resected colorectal cancer liver metastases (*n* = 21) and normal hepatic tissue specimens (*n* = 6) immunostained for annexin A1 (red). All sections were counterstained with 4′,6-diamidino-2-phenylindole (DAPI; blue). Original scale bars, 100 μm. Mann-Whitney test. Circles and squares indicate biological replicates, error bars indicate mean, whiskers indicate SE. *G*: ELISA for annexin A1 in serum from healthy blood donors (*n* = 30), patients with primary colon cancer (*n* = 30), and patients with liver metastases from colon cancer (*n* = 40). Kruskal-Wallis test with Dunn’s multiple comparison posttest. Circles, squares, and triangles indicate biological replicates, error bars indicate mean, whiskers indicate SE. *H*: overall survival of patients with colorectal adenocarcinoma who had an alteration in annexin A1 protein (alteration here means significant overexpression or underexpression). Log rank test. n.s., not significant; RPPA, reverse-phase protein array; TCGA, Tissue Cancer Genome Atlas.

We found that with the use of immunoblotting, annexin A1 was expressed by multiple cancer cell lines, with more prominent band intensity observed for colon cancer cell lines (MC38, HT-29, HCT116, LoVo) compared with pancreatic (Pan02) or lung (LLC) cancer cells (Fig. 5*A*). Bioinformatics-aided analysis of annexin A1 gene expression identified three studies where this molecule was significantly overexpressed in colorectal cancer samples compared with normal colonic mucosa (Fig. 5*B*). Annexin A1 exhibited a tendency to increase in human hepatic metastases compared with the uninvolved liver, based on a proteomics data set of five paired, resected specimens (59) (Fig. 5*C*). We further investigated annexin A1 concentrations using immunoblotting on an independent set of eight human-matched liver metastasis samples and observed its substantial abundance in tumor tissues (Fig. 5, *D* and *E*). Immunostaining for annexin A1 additionally revealed its membrane and pericellular ECM expression within human hepatic metastases, whereas associated unaffected liver displayed a dearth of positive staining, which was almost exclusively localized to the cell membrane (Fig. 5*F*). Metastasis tissues displayed variable levels of annexin A1, yet showing, on average, a larger positive staining area than uninvolved liver specimens (Fig. 5*F*). No significant difference, however, was observed with the evaluation of annexin A1 concentrations in serum of healthy blood donors, patients with primary colon cancer (Dukes’ stages A–C), and subjects with hepatic metastases from colon cancer (Dukes’ stage D; Fig. 5*G*). Intriguingly, a slight decrease in protein serum levels was observed, potentially suggestive of annexin A1 recruitment from the circulation to the tumor site, along with tumor progression. Finally, we questioned whether altered annexin A1 expression may influence prognosis of patients with colorectal cancer. The cBioPortal software demonstrated that annexin A1 predicted colorectal adenocarcinoma prognosis with a borderline significance (Fig. 5*H*; *P* = 0.07). Taken together, these data indicate that annexin A1 is frequently expressed in colorectal cancer ECM and may be important for progression of this disease.

#### Identification of cells producing annexin A1 in the TME.

**Fig. 6.**
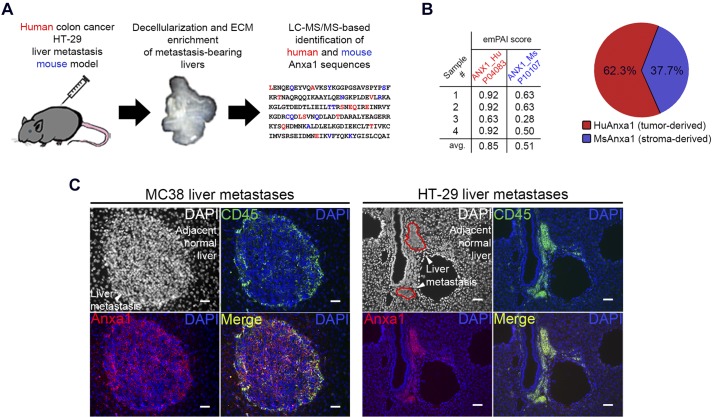
In the tumor microenvironment, annexin A1 is produced by both cancer and stromal cells. *A*: scheme illustrating the experimental pipeline. Human colon cancer cells HT-29 were injected intrasplenically into immunocompromised mice to establish hepatic metastases. Resultant liver tumors were decellularized and enriched, as described previously, with the following liquid chromatography tandem mass spectrometry (LC-MS/MS) analysis of human and mouse sequences. *B*: semiquantitative calculation of the exponentially modified protein abundance index (emPAI) (23) for estimation of a relative proportion of human-derived (i.e., cancer cell-derived; Hu) and mouse-derived (i.e., stroma-derived; Ms) annexin A1 (4 biological replicates per group). The pie chart reflects an averaged emPAI score for both human and mouse annexin A1. *C*: mouse liver metastases generated using MC38 and HT-29 cells were cryosectioned and stained for annexin A1 (red) and common bone marrow-derived cell marker CD45 (green). All sections were counterstained with 4′,6-diamidino-2-phenylindole (DAPI; blue). Original scale bars, 100 μm. ECM, extracellular matrix.

**Fig. 7.**
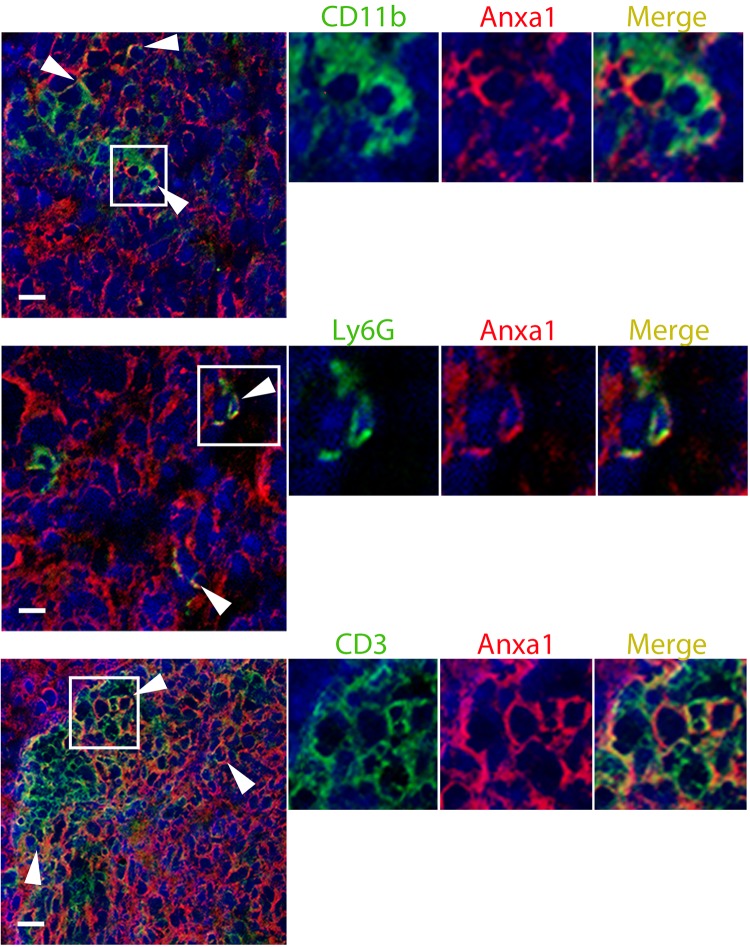
Immunostaining for annexin A1 (red) and immune cell markers CD11b, Ly6G, and CD3 (all green) displays their colocalization in the MC38 tumor microenvironment. All sections were counterstained with 4′,6-diamidino-2-phenylindole (DAPI; blue). Arrowheads indicate annexin A1 staining associated with immune markers. Original scale bars, 100 μm.

We sought to identify the cellular source of annexin A1 in the TME. To this end, we developed a murine liver metastasis model using human colorectal cancer cell line HT-29. With the exploitation of the advantage of the ability of LC-MS/MS to discriminate protein sequences of different species, we semiquantitatively determined tumor cell-derived (i.e., human) and stroma-derived (i.e., mouse) concentrations of annexin A1 using the exponentially modified protein abundance index (emPAI) (23) algorithm (Fig. 6*A*). Resultant emPAI scores are presented in Fig. 6*B* (*n* = 4 biological replicates). We found that approximately two-thirds of annexin A1 in the TME comes from cancer cells, whereas the remaining one-third is contributed from the host (Fig. 6*B*). Annexin A1 is known to regulate the immunity (10, 16), and thus we hypothesized that immune cells could contribute to the intratumoral annexin A1 levels. In mice, immunostaining for leukocyte common antigen CD45 revealed immune infiltration on the border of macrometastatic nodules, whereas micrometastases were completely infiltrated (Fig. 6*C*). We found colocalization of CD45 with annexin A1 in both MC38 and HT-29 liver metastases (Fig. 6*C*). To define immune cell populations contributing to annexin A1 production in these tumors, we performed immunophenotyping for a myeloid cell marker CD11b, neutrophil antigen Ly6G, and characteristic T cell molecule CD3. All of the above immune cell populations expressed annexin A1 to a certain extent (Fig. 7), suggesting that this protein is one of the immunohistochemical signature markers of major leukocyte types. Collectively, these findings indicate that most of annexin A1 in the TME is produced by the tumor, whereas cells of the myeloid lineage, including neutrophils and T cells, also express this molecule in some measure.

#### Annexin A1 is associated with S100-A11 in the liver metastatic ECM.

**Fig. 8.**
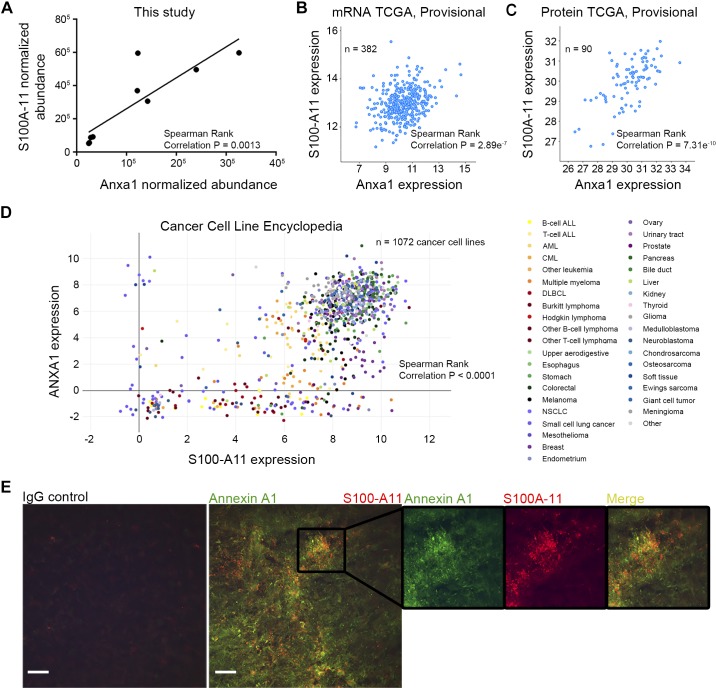
Annexin A1 is coexpressed with S100-A11 in liver metastasis extracellular matrix (ECM) and across other cancer types. *A*: normalized abundance of annexin A1 and S100-A11 in the quantitative proteomics data set from this study. Each circle indicates a replicate. *B* and *C*: coexpression of annexin A1 and S100-A11 in the Tissue Cancer Genome Atlas (TCGA) data set (colorectal adenocarcinoma), as assessed by mRNA (*B*) and protein (*C*) levels. Each circle represents a patient. *D*: coexpression of annexin A1 and S100-A11 in the Cancer Cell Line Encyclopedia data set. Each circle represents a human cancer cell line. ALL, acute lymphoblastic leukemia; AML, acute myelogenous leukemia; CML, chronic myelogenous leukemia; DLBCL, diffuse large B cell lymphoma; NSCLC, nonsmall cell lung carcinoma. *A–D*: Spearman rank correlation was measured. *E*: immunostaining for annexin A1 and S100-A11 in the decellularized and cryosectioned ECM from MC38 murine liver metastasis. Negative IgG control staining is shown to the left. Original scale bars, 100 μm.

Annexins frequently bind to S100 proteins to form functionally active complexes (46). Annexin A1 has a strong affinity to S100-A11 (44), and our proteomics list revealed that both annexin A1 and S100-A11 overexpressed in metastasis ECM in similar molar concentrations (Fig. 3). We documented a positive correlation between protein abundances of annexin A1 and S100-A11 across samples analyzed by proteomics (Fig. 8*A*). In keeping with these findings, expression of annexin A1 strongly correlated with S100-A11 expression in the Tissue Cancer Genome Atlas data set of colorectal adenocarcinoma patients, both at mRNA and protein levels (Fig. 8, *B* and *C*). Furthermore, mRNA levels of annexin A1 and S100-A11 positively correlated in 1,072 comprehensively characterized human cancer cell lines (Fig. 8*D*) from the Cancer Cell Line Encyclopedia data set (17), confirming that this association is also relevant for cancer types other than colorectal. To examine if these molecules may be colocalized specifically within the ECM, we sectioned the ECM of murine hepatic metastases with the following costaining. Both proteins were highly abundant in the metastatic ECM, confirming the results obtained during LC-MS/MS analysis (Fig. 8*E*). Strikingly, we observed multiple areas where the annexin A1 signal was tightly associated with S100-A11, suggesting the presence of the annexin A1-S100-A11 complex within the ECM (Fig. 8*B*).

#### Annexin A1 knockdown inhibits proliferation and promotes migration of cancer cells.

**Fig. 9.**
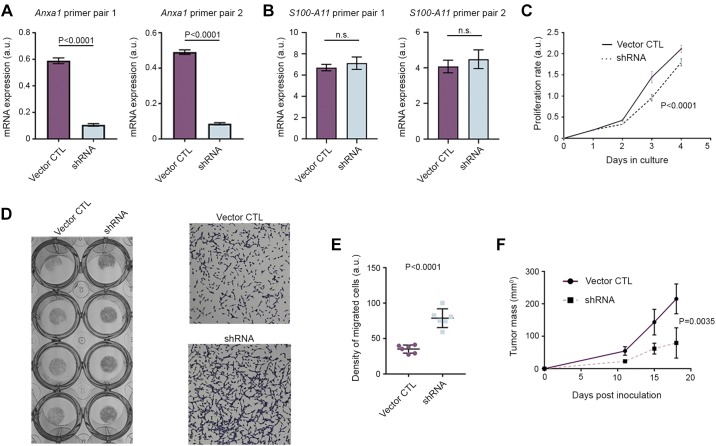
Annexin A1 reduces proliferation and promotes migration of cancer cells in vitro and diminishes xenograft growth in vivo. *A* and *B*: PCR evaluation of *Anxa1* (*A*) or *S100-A11* (*B*) transcripts in Lewis lung carcinoma (LLC) cells transfected with empty vector [control (CTL)] or shRNA targeting *Anxa1*. Mann-Whitney *U*-test was used. *C*: proliferation rate of control and *Anxa1*-deficient cells. Two-way ANOVA was used. *D*: photograph of the Boyden chamber (left) and microphotographs (right) of crystal violet-stained control and *Anxa1*-deficient LLC cells subjected to 48 h Transwell migration assay. *E*: density quantification of migrated cells in microphotographs from the experiment in *D*. Each circle and square represents 1 field of view. Mann-Whitney *U* test was used. *F*: tumor growth curves of control and *Anxa1*-deficient LLC cells implanted into flanks of C57BL/6 mice. Two-way ANOVA was used. a.u., arbitrary units; n.s., not significant.

To gain a functional insight on annexin A1 biology in the context of cancer, we performed a stable knockdown of this molecule using shRNA lentiviral transfection. Transfected cancer cells reached ~80–85% inhibition of *Anxa1* mRNA production, as assessed by two different primer pairs (Fig. 9*A*). We then questioned if *S100-A11* gene expression can be altered upon *Anxa1* knockdown but found no alteration of mRNA levels compared with vector control cells (Fig. 9*B*). This finding suggested that coexpression of these two proteins is unlikely to be a result of a positive feedback loop, where one molecule enhances the expression of the other. *Anxa1*-deficient cells grew 30–35% slower compared with vector control cells (Fig. 9*C*); however, they exhibited an increased rate of Transwell migration compared with vector control cells (Fig. 9, *D* and *E*). To investigate whether *Anxa1* knockdown impacts on tumor cell proliferation in vivo, we injected them subcutaneously into flanks of C57BL/6 mice (*n* = 5 mice per group). We identified that *Anxa1-*deficient cells grew as xenografts, substantially slower than their control counterparts (Fig. 9*F*).

## DISCUSSION

Liver metastases are a dangerous consequence of colorectal cancer, commonly resulting in morbidity and death. Because of progress in anesthetic and surgical techniques, hepatic surgery has become a treatment of choice for patients present with liver metastatic disease; however, the disease-free survival does not exceed 25% (15). Identification of molecular phenotypes of colorectal cancer revealed the significance of its mesenchymal subtype, characterized by the worst relapse-free and overall survival, with key hallmarks being excessive ECM deposition, stromal infiltration, and transforming growth factor (TGF)-β activation (18). The ECM influences TGF-β signaling activation by releasing the biologically active TGF-β molecule from its latent complex, a process modulated by integrins αvβ6, αvβ8, and α5β1 and certain matrix proteases, including matrix metalloproteinases 2 and 9 and bone morphogenetic protein 1 (22). Proteomics analysis of the matrisome from human colorectal cancer liver metastases revealed a strong over-representation of the TGF-β pathway (59), whereas inhibition of TGF-β signaling substantially diminished murine experimental hepatic metastases from colon cancer (61, 64). Thus, the matrisome and its alterations are involved in important aspects of colorectal metastatic disease, yet the topic remains insufficiently studied.

The purpose of this study was to examine MC38 liver metastatic ECM compared with that of intact, disease-free liver. We did not investigate any potential matrisome changes to the host liver away from the metastatic sites (such as conditionally unaffected tissues adjacent to metastasis). To address this aim, we used an MS-aided approach. However, isolation and purification of the ECM are challenging due to several reasons. First, matrix proteins represent high molecular mass and highly crosslinked molecules; these features greatly limit their denaturation and solubilization. Second, lysis of cells during ECM extraction releases intracellular components into the extracellular space, thus leading to contamination by nucleic acids and proteins with high affinity to matrix molecules. Third, because of lengthy and laborious protocols used for ECM extraction, the washout and subsequent loss of matrisome proteins are likely, resulting in sample analysis distortion. All of these issues have been addressed by our optimized workflow of mild detergent solubilization, followed by gentle biochemical enrichment. Our method has previously been successfully used for matrisome analysis by proteomics (59).

In this study, we have found 27 proteins differentially expressed between MC38 hepatic metastases and normal mouse liver. Although significantly downregulated molecules represent probable liver-specific ECM components, overexpressed proteins are of great interest; some of them were previously linked to the development and progression of colorectal cancer, including versican (9, 10a), osteonectin (1, 51), galectins (3, 41, 48), and protein of the S100 family (24, 49). Interestingly, a combination of 13 overexpressed proteins significantly predicted overall survival from colorectal adenocarcinoma but not cancers of the breast and pancreas, both characterized by substantial ECM influence. This suggests our protein combination as specifically relevant for colorectal cancer progression.

Annexin A1 is little studied in the context of colorectal cancer, and its ECM localization is not recognized. It was previously reported that isolated bovine cartilage contained annexin A1 bound to collagen XI (5), a small regulatory molecule associated with altered TGF-β signaling, metastasis, and poor survival (8). Here, we found annexin A1 overexpressed in colon cancer cell lines compared with cancer cells of other origin and over-represented in human primary colorectal lesions as well as hepatic metastases compared with their adjacent healthy tissue counterparts. Immunostaining of metastatic hepatic tissues revealed extracellular, pericellular, and membrane-associated expression patterns of annexin A1, whereas unaffected liver tissues exhibited mostly membrane-bound localization of this molecule. Serum annexin A1 levels were unchanged in metastatic colorectal cancer subjects compared with healthy blood donors. Findings presented here are consistent with the data obtained by Su et al. (50), reporting annexin A1 overexpressed in colorectal cancer patients with the *K-Ras* mutation. Similarly, Ydy et al. (57) observed higher levels of annexin A1 in colon but not rectum cancer tissue specimens compared with normal margin tissue.

With regard to its function in liver metastases, little is known about annexin A1. In human colon cancer cells, annexin A1 inhibited NF-κB by directly binding to its p65 subunit (62). Bioactive NH_2_-terminal peptides of annexin A1 inhibited NF-κB and the growth of SW480 colon cancer cell xenografts in nude mice (62). In our study, annexin A1 and S100-A11 were simultaneously overexpressed in the ECM isolated from mouse hepatic metastases, displaying a significant positive correlation between their concentrations across all samples. In the decellularized metastatic ECM, annexin A1 immunostaining was tightly associated with the S100-A11 signal, and close proximity between these molecules is consistent with coimmunoprecipitation studies where these two proteins formed a complex (56). Importantly, annexin A1 functionality greatly depends on whether it is present in a free form or in complex with S100-A11 (11). For example, disruption of the annexin A1/S100-A11 complex enhanced the migration and clonogenic growth of ovarian cancer cells by modulating epithelial growth factor signaling (42). Similarly, epithelial growth factor signaling was modulated by the annexin A1/S100-A11 complex tethering a subpopulation of membrane contact sites between the endoplasmic reticulum and endocytic organelles (12). The presence of the annexin A1/S100-A11 complex in the ECM has not previously been documented.

Our studies reveal annexin A1 mostly deriving from the cancerous tissue, whereas tumor-infiltrating leukocytes produce approximately one-third of this molecule in the TME. Annexin A1 is a major player in plasma membrane repair (32), and it could be speculated that immune cells secrete this molecule in attempt to fix plasma membrane damage caused by their infiltration into dense, rigid stroma. Follow-up studies should be aimed to address this hypothesis.

To conclude, here, we quantitatively characterized the matrisome of MC38 murine hepatic metastases. Findings presented here suggest the importance of annexin A1 in altering migration of cancer cells as well as their proliferation, both in culture and as subcutaneous xenografts. Our results point to annexin A1 as a putative biomarker or therapeutic target in liver metastases from colorectal cancer.

## GRANTS

Support for this study has been provided by CRUK funding to the Oxford Institute for Radiation Oncology (C5255/A15935) and CRUK/EPSRC Oxford Cancer Imaging Centre (C5255/A16466).

## DISCLOSURES

No conflicts of interest, financial or otherwise, are declared by the authors.

## AUTHOR CONTRIBUTIONS

A.E.Y., D.Y., and R.J.M. conceived and designed research; A.E.Y., S.Y.L., and B.M.K. performed experiments; A.E.Y. and R.F. analyzed data; A.E.Y., S.Y.L., and A.N.G.-W. interpreted results of experiments; A.E.Y. prepared figures; A.E.Y. drafted manuscript; R.J.M. edited and revised manuscript; R.J.M. approved final version of manuscript.

## Footnotes

1 Supplemental Material for this article is available online at the Journal website.
